# Tetanus toxin C-fragment protects against excitotoxic spinal motoneuron degeneration *in vivo*

**DOI:** 10.1038/s41598-018-35027-w

**Published:** 2018-11-08

**Authors:** Citlalli Netzahualcoyotzi, Ricardo Tapia

**Affiliations:** División de Neurociencias, Instituto de Fisiología Celular, Universidad Nacional Autónoma de México, 04510 Ciudad de México, Mexico

## Abstract

The tetanus toxin C-fragment is a non-toxic peptide that can be transported from peripheral axons into spinal motoneurons. In *in vitro* experiments it has been shown that this peptide activates signaling pathways associated with Trk receptors, leading to cellular survival. Because motoneuron degeneration is the main pathological hallmark in motoneuron diseases, and excitotoxicity is an important mechanism of neuronal death in this type of disorders, in this work we tested whether the tetanus toxin C-fragment is able to protect MN in the spinal cord *in vivo*. For this purpose, we administered the peptide to rats subjected to excitotoxic motoneuron degeneration induced by the chronic infusion of AMPA in the rat lumbar spinal cord, a well-established model developed in our laboratory. Because the intraspinal infusion of the fragment was only weakly effective, whereas the i.m. administration was remarkably neuroprotective, and because the i.m. injection of an inhibitor of Trk receptors diminished the protection, we conclude that such effects require a retrograde signaling from the neuromuscular junction to the spinal motoneurons. The protection after a simple peripheral route of administration of the fragment suggests a potential therapeutic use of this peptide to target spinal MNs exposed to excitotoxic conditions *in vivo*.

## Introduction

Motoneuron (MNs) diseases are a heterogeneous group of disorders characterized by muscle weakness and/or spastic paralysis, which result from the selective degeneration of lower motor neurons and/or upper motor neurons, respectively^1^. Amyotrophic lateral sclerosis (ALS) is the most common and fatal form of these disorders, with a global incidence of ~3 cases/100,000 population^2^. Although several mechanisms (oxidative stress, mitochondrial dysfunction, endoplasmic reticulum stress, protein aggregation, RNA metabolism, neuroinflamation, etc.) have been suggested to explain the causes underlying the selective degeneration of MNs, the glutamate-induced excitotoxicity is considered as one of the major pathophysiological factors participating in the development of the disease^3,4^.

Our group has shown that the excitotoxicity produced by chronic infusion of α-amino-3-hydroxy-5-methyl-4-isoxazolepropionic acid (AMPA) in the rat lumbar spinal cord leads to MN death, accompanied by intense astrogliosis, that results in progressive motor behavior alterations and finally irreversible paralysis of the rear limbs^5–7^. Importantly, this well-established model of progressive spinal MN degeneration *in vivo* does not depend on genetic alterations, as is the case in the vast majority of ALS (for a recent ample review see^8^). Furthermore, as we have shown and discussed previously, the neuropathological changes observed in the spinal cord^9^, as well as several structural and functional mitochondrial alterations that we have described in our model^10,11^ are similar in several aspects to those found in the tissue of ALS patients.

Among the many compounds that have been tested to prevent or delay the progress of neurodegeneration in ALS, growth factors, including the neurotrophin family, have been considered as a potential beneficial strategy^12–15^. Nonetheless, clinical testing of these growth factors in ALS have been unsuccessful^16–18^, owing in part to the poor pharmacokinetic profiles of these factors, including rapid enzymatic inactivation, fast clearance process, sequestration, and potential immunogenicity, which complicate reaching target sites inside the central nervous system^19^. Hence it is of importance to look for alternative strategies that permit or facilitate the transport of the factors to reach MNs and exert their neurotrophic signaling.

The tetanus toxin produced by the anaerobic bacteria *Clostridium tetani* is a single-chain polypeptide of approximately 150 kDa, which is post-translationally nicked to form the active isoform conformed of a light chain (~50 kDa) and a heavy chain (~100 kDa) linked by a disulfide bond. The catalytic and neurotoxic domain of the toxin resides in the light chain, while the translocation and receptor-binding domains are present in the heavy chain. The heavy chain has been cleaved with papain into two parts (~50 kDa each), consisting of the tetanus toxin carboxyl-fragment (TTC) and the N-terminal portion of this chain^20,21^. The TTC does not have the toxic effects of the native toxin^22,23^, but maintains the capacity of membrane binding^24–26^, internalization, and retrograde transport^27^ with preferential localization in MNs, as demonstrated by studies *in vitro*^21,28^ and *in vivo*^29–31^. Furthermore, this fragment activates neurotrophin-regulated signaling pathways in a Trk receptor-dependent manner *in vitro*^32–34^ and has neuroprotective effects in primary cerebellar granule cell cultures exposed to toxic low concentration of potassium or treated with 1-methyl-4-phenylpyridinium^32,35^, as well as in animal models of cerebral ischemia^36^ and Parkinson’s disease^37^. TTC also improved MN survival in spinal cord organotypic cultures exposed for short time periods to glutamate^38^. Moreover, gene therapy to induce recombinant molecule linked TTC has shown promising neuroprotective results in the mutant SOD1-G93A murine model of familial ALS^39^. However, the efficacy of the fragment to protect against excitotoxic degeneration of spinal MN in *in vivo* has not been studied, and this is the aim of the present work.

For this purpose, we tested the effect of the intraspinal and the intramuscular (i.m.) administration of the fragment, and we found that the i.m. injection significantly protected against the neurodegeneration, motor deficits and rear limb paralysis in our previously described model of chronic AMPA-induced excitotoxity^5–7^. Furthermore, we determined that TTC i.m. treatment increases neurotrophic receptor tyrosine kinase 1 (TrkA) phosphorylation (Y490) in spinal MNs and that the unspecific blockade of Trk receptors in the neuromuscular junction (NMJ) partially prevents the TTC-induced neuroprotective effects. These results are the first demonstration of a protective action of TTC against excitotoxic neurodegeneration in the spinal cord *in vivo* and that this action occurs mainly after the intramuscular administration of the fragment.

## Methods

Adult male Wistar rats (280–300 g) were used in all the experiments and were handled in accordance with the Official Mexican Regulation concerning the Laboratory Animal Welfare (NOM-062-ZOO-1999) and with approval of the Institutional Committee for the Care and Use of Laboratory Animals of the Instituto de Fisiología Celular, Universidad Nacional Autónoma de México (Approval No. RTI21-14). Rats were housed in a laboratory environment with a 12 h light/dark cycle and with food and water ad libitum along the entire experiment. All efforts were made to avoid unnecessary suffering of the animals.

### Surgery and drug administration

The implantation of the osmotic minipumps and intraspinal cannula was carried out essentially as previously described^5^, and therefore only a brief description will be given. AMPA concentration was chosen on the basis of this previous work.

**Figure 1:**
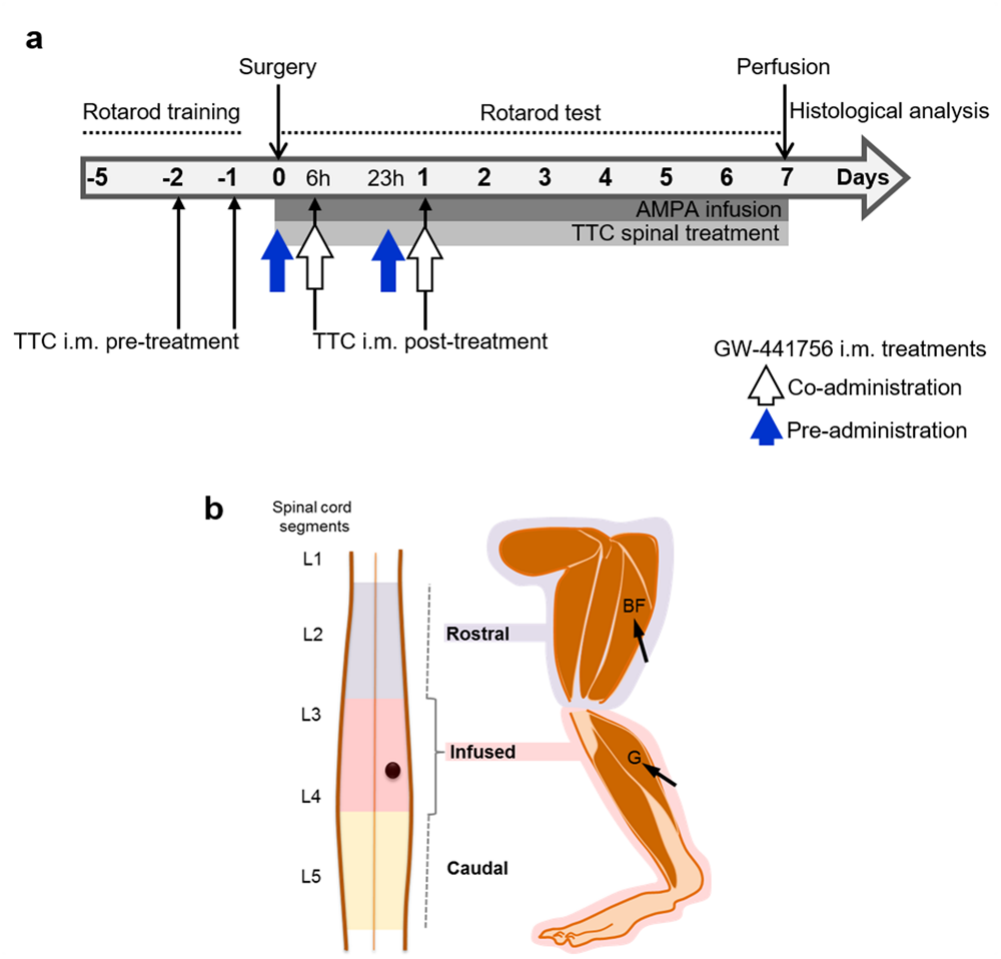
Experimental design. Scheme (**a**) indicates the timing of the rotarod training and test, and the duration of the continuous spinal infusion of AMPA and TTC (grey horizontal bars). Black thin arrows indicate the timing of TTC i.m. injections before (TTC i.m. pre-treatment) or after (TTC i.m. post-treatment) surgery. The TrkA blocker GW-441756 was injected i.m. at the same time of TTC (co-administration, white arrows) or before TTC (pre- administration, blue arrows). All i.m. treatments were made bilaterally in both the gastrocnemius (G) and biceps femoris (BF) muscles shown in (**b**), as related to the infused (pink) and rostral (blue) regions of the spinal cord. These regions are intensively injured by AMPA and were analyzed separately by histology (Fig. 3b). The black dot represents the position of the probe in the spinal cord.

RS-AMPA (Tocris, Ellisville, MO) was dissolved in 0.1 M phosphate buffer (PB), pH 7.4. The osmotic minipumps (Alzet model 2004, capacity ~250 µL, flow rate 6 µl/day) were filled with vehicle as a control, 1 mM AMPA, TTC at the concentration indicated below, or AMPA + TTC, and they were incubated in sterile isotonic saline solution at 37° for 48 h for stabilization before the implantation. The day of surgery rats were anesthetized with isoflurane 1–2% in a 95% O_2_ + 5% CO_2_ mixture and placed in a stereotaxic spinal unit. After shaving and disinfection of the skin an incision was made on the back of the animal. The lumbar region was carefully exposed and the muscles surrounding the T13 vertebra were retracted. The spinous process of this vertebra was lowered and on this site a stainless-steel screw (3.7 mm long, 1 mm diameter) was inserted to anchor the implant. A 1–2 mm diameter hole was drilled at the same vertebrae and a small cut of the meninges was made to insert a borosilicate glass probe (1 mm long, 50 µm I.D. × 80 µm O.D., VitroCom Inc.) into the right dorsal horn of the spinal cord (between L3–L4 spinal segments, Fig. 1b). The other end of the cannula was connected through a plastic tubing (1.7 cm long) to the osmotic minipump, which was implanted subcutaneously in the back of the rat. The probe and the screw were fixed to the bone with dental acrylic. At the end of the surgery, the skin incision was closed with surgical stainless-steel clips and rats received a single i.m. dose of penicillin (50 U). After recovering from anesthesia animals were kept in individual cages and it was verified that there were no motor alterations because of the surgical procedure.

TTC was obtained commercially (Santa Cruz Biotechnology, Dallas, TX), dissolved before the experiment in 0.1 M PB and administered by means of two different routes: direct spinal infusion or i.m. injection. For the spinal treatment TTC, alone or mixed with AMPA, was infused through the minipump at a 20 nM concentration, which was chosen on the basis of the results of experiments *in vitro*^33,34^. As indicated in Fig. 1a, the TTC spinal infusion lasted seven days and, as a result, a total amount of ~42 ng of the fragment were infused into the spinal cord of each rat.

On the basis of the rear limb-paralysis sequence in our model (distal to proximal)^5^, we decided to inject the fragment into the gastrocnemius and the biceps femoris muscles (Fig. 1b), corresponding to distal and proximal muscles of the rear limbs affected by the neurodegeneration of the lumbar spinal MNs. As indicated in Fig. 1a, we injected TTC following two different protocols: pre-treatment injections were made 24 h and 48 h before the surgery, and post-treatment injections, 6 h and 24 h after the implantation. All injections (100 µL each) were made bilaterally into the belly of the two muscles mentioned. The final dose of each treatment was 1.3 µg/kg (~400 ng/rat). This dosage is one of the lowest previously reported to be effective^40^.

**Figure 2:**
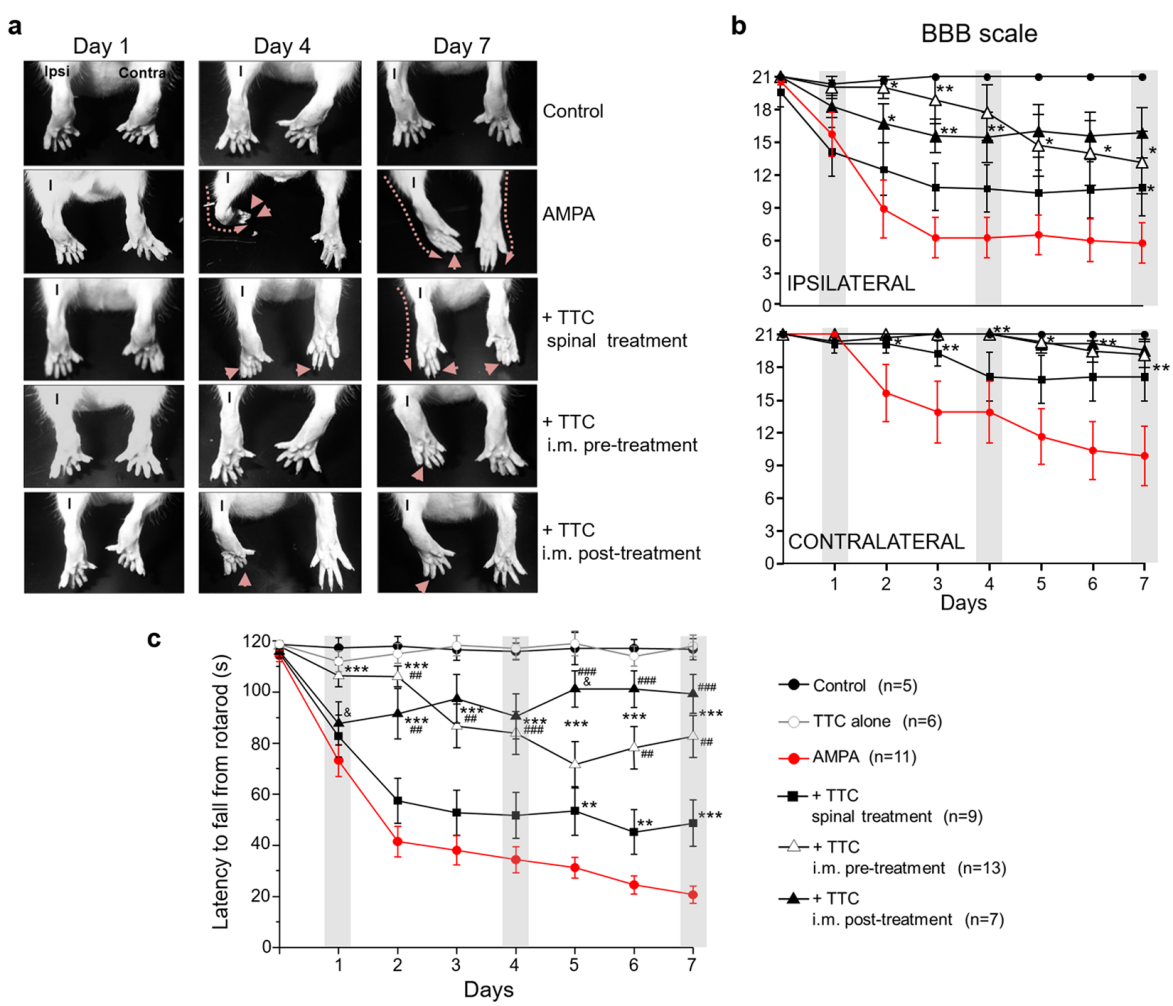
Progressive motor alterations in rear limbs induced by AMPA and protection by TTC. (**a**) Representative posterior views of the rear limbs (I, ipsilateral) at the beginning (day 1), the middle (day 4) and last day (day 7) of the experiment show the alterations in the position of the rear limb (discontinuous lines) and phalanges (arrowheads). (**b**) Motor function of ipsilateral and contralateral rear limbs assessed by the BBB scale. A value of 21 points refers to the natural and correct movement of the rear limb, whereas 0 point denotes a total paralysis of the extremity. (**c**) Motor performance assessed by the rotarod test. Three trials/day/rat were assessed on the rotarod test. The spinal treatment was less effective. Grey bars in the graphics highlight the initial, middle and last day of infusion shown in (**a**). Data are mean values ± SEM for the number of animals shown in parentheses. The TTC alone group includes 3 rats treated with i.m. TTC injection and 3 with spinal infusion. *p < 0.05, **p < 0.01, ***p < 0.001 vs AMPA. ^##^p < 0.01, ^###^p < 0.001 vs spinal and ^&^p < 0.01 vs pre-treatment.

**Figure 3:**
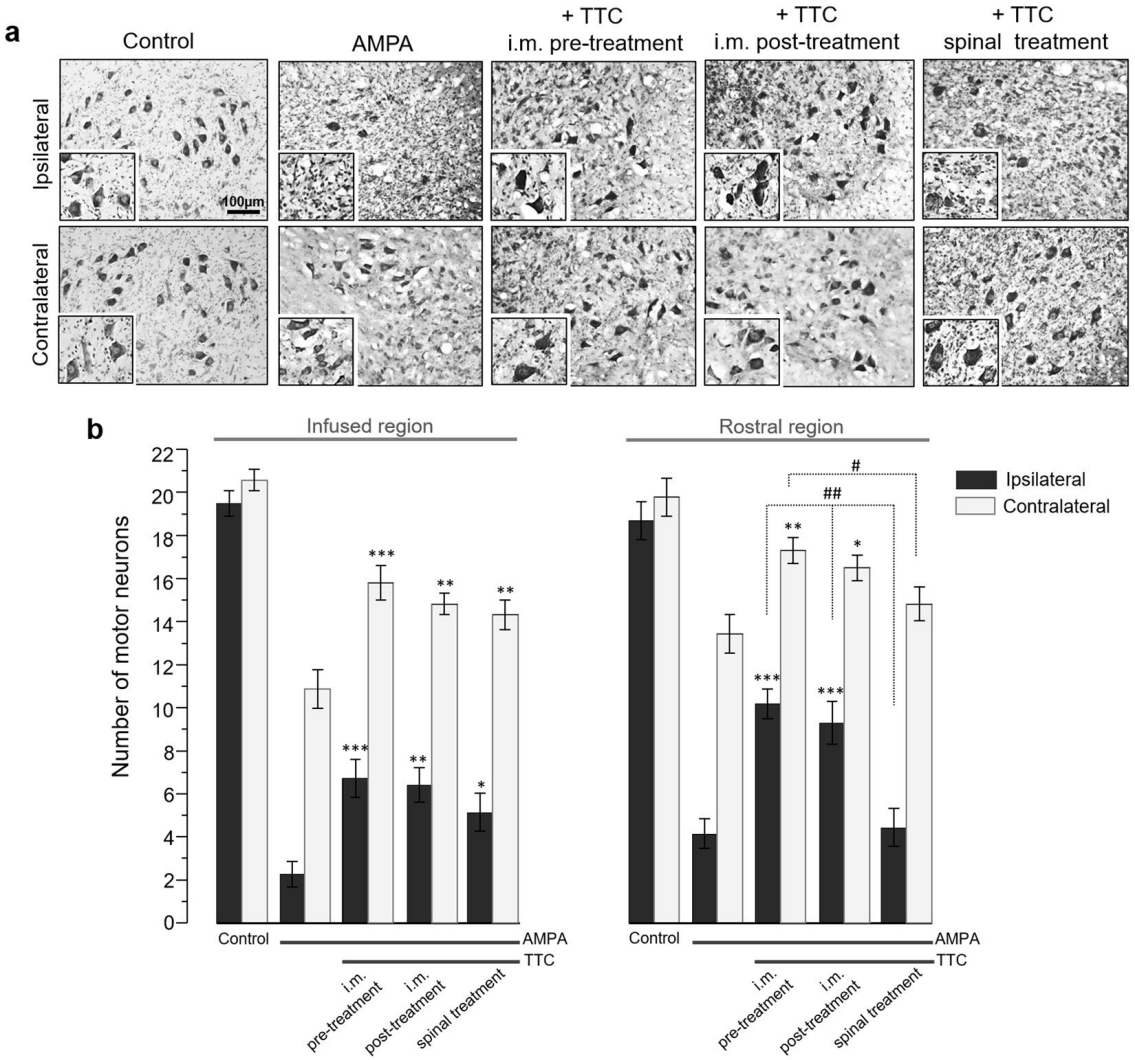
TTC treatment protects against the spinal MN degeneration induced by chronic excitotoxicity in the spinal cord. This histological analysis was made in the spinal cord tissue of rats treated as indicated, seven days after minipump implantation. (**a**) Representative micrographs of Nissl-stained sections of the infused lumbar spinal cord region (quantitative analysis in (**b**)), and their corresponding 150% magnification in the inset. (**b**) Number of healthy MNs in the ventral horns in the infused spinal cord region and in the adjacent rostral zone. AMPA elicited almost complete loss of spinal MNs and TTC, mainly after i.m. administration, prevented this damage in both the infused and the rostral regions. Fifteen histological slices/rat/region were analyzed. Data are mean values ± SE for 5 (control), 10 (AMPA), 11 (TTC pre-treatment), 7 (TTC post-treatment) and 8 (TTC spinal infusion) rats. *p < 0.05, **p < 0.01, ***p < 0.001 vs corresponding side of the AMPA group in the corresponding region (infused or rostral). ^##^p < 0.01, ^##^p < 0.01 vs corresponding side of the spinal treatment.

In other experiments we dissolved GW-441756 in DMSO (Sigma, St. Louis, MO), and it was administered i.m. (430 µg/kg, ~130 µg/rat) simultaneously with the TTC (co-administration), or 6 h before the first injection of TTC and one h before the second injection (pre-administration) (white and blue arrows in Fig. 1a, respectively). We also evaluated the i.m. administration of DMSO alone as a vehicle control, and DMSO + GW-441756 alone, as controls. In these experiments, some rats treated with AMPA alone and with AMPA + TTC post-treatment were included in parallel and the data were grouped with other rats similarly treated. Therefore, the results of these two groups are the same in Figs 2b,c, 3b and 6.

### Assessment of motor function

Five days before surgery rats were trained to reach 120 s on the rotarod test (Columbus Instruments, Columbus, OH), with rotation starting at 10 rpm and accelerated at 0.2 rpm/s. The rats that did not fully complete the rotarod training were discarded from the experiment before surgery. Three trials/day/rat were assessed on the rotarod from the day of surgery and until the seventh day, when animals were anesthetized and perfused for histology (Fig. 1a). In addition, we placed the animals over a flat surface to rate the movement of each one of the rear limbs based on the BBB scale described in ref.^41^ (details of the BBB scale are shown in Supplementary Table 1). Likewise, holding up the tail we daily photographically recorded the changes in the position of the rear limbs along the experiment.

### Histology and immunohistochemistry

For the histological and immunohistochemical analyses of the spinal cord, rats were transcardially perfused seven days after osmotic pump implantation (unless indicated otherwise), with 0.9% saline solution followed by 4% paraformaldehyde in PB pH 7.4. Spinal cords were removed, post-fixed 48 h at 4 °C and dehydrated with sucrose solutions (up to 30%). Transverse sections (40 μm thick) of the spinal cords were obtained in a cryostat. Sixty serial slices were obtained from the site of the cannula infusion and sixty from the immediate region rostral to the infused zone. These are referred in Results as the infused and rostral region, respectively. Alternate sections were stained with cresyl violet or immunostained as indicated below. The Nissl-stained slices were used for the assessment of morphologically healthy MNs in the ipsilateral and contralateral ventral horns. Large neurons with a soma diameter >20 μm and distinguishable nucleus, similar in appearance to those of the control and intact rats (Fig. 3a) were manually counted. Fifteen histological slices/rat/region were analyzed.

For immunohistochemistry, we used the neurofilament protein SMI-32 and GFAP as markers of neurons and astrocytes, respectively. We also determined the location of the TrkA receptor but in these experiments animals (n = 4 per group) were fixed four days after surgery (or four days after the first i.m. injection in the TTC-alone group), in order to detect and analyze a sufficient number of MNs, before the occurrence of the extensive AMPA-induced damage. In brief, the free-floating sections were blocked with 5% bovine serum albumin in phosphate buffer saline (PBS)–Triton X-100 (0.3%) for 2 h, and then incubated with the corresponding primary antibody: mouse polyclonal anti-SMI-32 (1:1000, Covance), chicken polyclonal anti-GFAP (1:1000, Abcam) and rabbit polyclonal anti-phosphorylated TrkA (phospho-Y490) (10 µg/ml, Abcam) for 48 h at 4 °C. Then the slices were washed 4 × 15 min in PBS–Triton X-100 and incubated with the secondary antibody for 2 h at room temperature. We used goat anti-mouse Texas-Red (Invitrogen), goat anti-chicken FITC (Novex), and for confocal microscopy we worked with donkey anti-mouse Alexa546 (Invitrogen) and goat anti-rabbit Cy5 (Life Technologies). Finally, sections were washed and mounted on silane-covered slides and coverslipped with fluorescent mounting medium (DAKO). The cross-reactivity in the immunofluorescent technique was excluded by control slices incubated in the absence of primary antibodies. There was no immunostaining in these controls.

For the quantitative analysis of fluorescence images all the pictures analyzed for each parameter were acquired using the same settings. The immunostaining for SMI-32 and GFAP was visualized under an epifluorescence Olympus microscope. The mean fluorescence intensity (MFI) labeling was determined using epifluorescence images of the channel corresponding only to GFAP in the ipsilateral ventral horn. We first verified that there was not threshold over/undersaturated pixels. Then the MFI was measured and normalized by area. Three histological slices/rat were analyzed (n = 3 per group). SMI-32 and TrkA receptor (phospho-Y490) were observed by confocal microscopy (LSM 710-Zeiss). The merged images are the maximum intensity projection obtained from Z-stack analysis. The corrected total cellular fluorescence (CTCF) for TrkA in the spinal MNs was made using the maximum intensity projection of the Z-stack images for TrkA (phospho-Y490) alone, and in accordance with the formula previously reported: CTCF = integrated density − (area of selected cell × mean fluorescence of background readings)^42^. Five histological slices/rat were analyzed (n = 4 per group). Merged images, MFI and CTCF were obtained with ImageJ/FIJI (NIH, Bethesda, MD).

### Statistical analysis

The time on the rotarod and the number of MNs was analyzed by ANOVA followed by Tukey post hoc test. To analyze the MFI and CTCF we used Kruskal-Wallis, Dunn’s Multiple Comparison post hoc test. A value of p < 0.05 was considered statistically significant. The statistical analysis was performed using Prism 5 Software (San Diego, CA).

## Results

We have studied the motoneuron degeneration and consequent motor alterations induced by the chronic infusion of AMPA, by means of osmotic minipumps, in the rat lumbar spinal cord, and the protective effect of TTC, when co-infused with AMPA and when injected i.m.

### TTC i.m. administration exerts better protection than the spinal infusion

**Figure 4:**
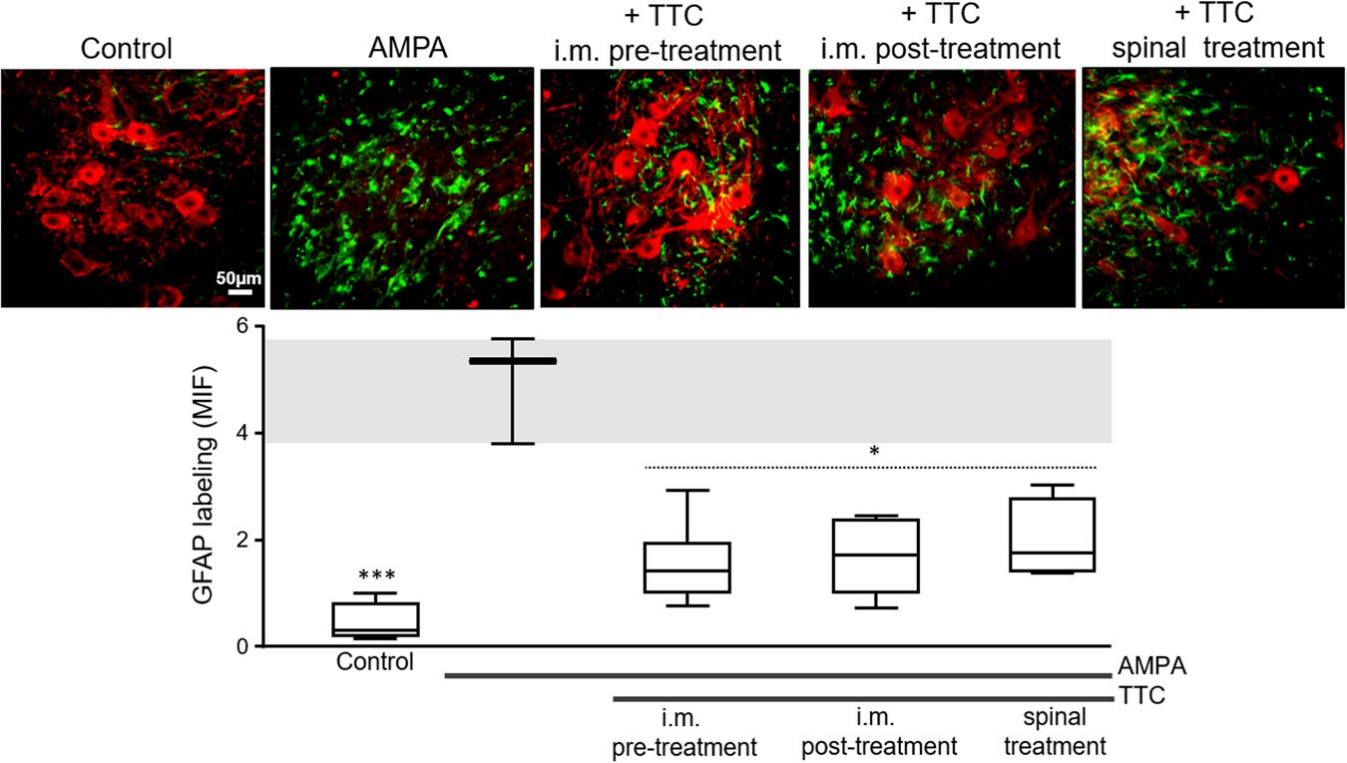
TTC attenuates the AMPA-induced astrogliosis. Representative epifluorescence images of SMI-32 (red) and GFAP (green) immunohistochemistry of the ipsilateral ventral horns at the infused spinal cord region. AMPA elicited a notable astrogliosis that was reduced by either i.m. or spinal TTC. These effects were similar in the contralateral ventral horn. The graphic in the lower panel shows the mean intense fluorescence (MIF) for GFAP labeling in the ipsilateral ventral horn of the infused region. The box-and-whisker plot includes the data obtained from the analysis of 3 histological slices/rat (n = 3 per group). The boxes represent the 1st and 3rd quartile around the median. The whiskers above and below the box show the locations of the minimum and maximum values. The grey horizontal area highlights the maximum and minimum value for the AMPA group. *p < 0.05, ***p < 0.001 vs the AMPA group.

The control rats infused in the spinal cord or i.m. administered with vehicle or TTC alone did not exhibit motor dysfunctions or histological damage at any time (Figs 2, 3 and 4). The continuous infusion of 1 mM AMPA triggered alterations in the position of the phalanges of the ipsilateral rear limb, which started on the first day and gradually progressed distal-caudally to the entire limb. With a short delay the same effect encompassed the contralateral side, resulting at the end of the experiment in a complete paralysis of the ipsilateral paw, with a BBB score ~6 (20 points meaning normal movement)^41^ and partial loss of the locomotor activity on the contralateral side (BBB score ~10) (Fig. 2a,b). The progress of these alterations is clearly observed in the rotarod test and, as described in other articles of our group, is also manifested in a grip paw endurance test and in a decrease of rear limbs grip strength^5–7^. Whereas control rats remained in the rod for the total test period of 120 s, the score of animals treated with AMPA was 73.2 ± 6.2 s at day one, and progressively decreased until 20.7 ± 3.4 s on the seventh day (Fig. 2c).

The protective effect of TTC was first evaluated by its continuous spinal infusion together with AMPA (Fig. 1a). At the end of the experiment, these animals were not able to control the ipsilateral phalanges (BBB score ~11) but there was still control of the contralateral ones (BBB score ~17) (Fig. 2a,b). Their rotarod performance was not different as compared with AMPA alone until day 4, but on the last three days these animals showed a slight although significant improvement in their motor performance, scoring 53.4 ± 9.5, 45.2 ± 8.7 and 48.7 ± 9.1 s, respectively (Fig. 2c). Notably, the i.m. administration of TTC exerted a much better protection: both pre- and post-treatments almost totally prevented the AMPA-induced alterations in the phalanges of rear limbs (ipsilateral minimum BBB points ~14, contralateral no different from control) (Fig. 2a,b). The protection exerted by the pre-treatment was highly significant along the entire duration of AMPA infusion, since the time on the rotarod was 106.4 ± 4.3 s at day one and 82.6 ± 8.2 s at the last day, close to the control score. The post-treatment after 6 and 24 h of the osmotic pump implantation was also effective, but not in day one (24 h post-surgery) when the animals of this group scored 87.7 ± 8.4 s in the rotarod test (not different from AMPA group). It was until day two that the performance of these animals improved, scoring better times until achieving 99.3 ± 7.6 s in day seven (Fig. 2c). In summary, AMPA induced a progressive and intense alteration in both extremities, whereas i.m. TTC partially prevented this effect, with corresponding higher scores in both the BBB scale and the rotarod test.

We have previously shown^5^ that under our experimental conditions AMPA causes degeneration mainly in the infused region of the spinal cord, but it also causes MN death in immediate rostral region; therefore, in this work we examined histologically both zones (Fig. 1b). The histological examination (Fig. 3) revealed that after seven days of surgery control rats exhibited ~20 MNs per section with normal morphology. At the concentration used AMPA caused a severe damage in the ipsilateral ventral horn of the infused region, where we recorded only 2.3 ± 0.6 healthy MNs (88% loss as compared to the control rats) and 11.1 ± 1.0 (47% loss) in the contralateral side. This damage was accompanied by intense reactive astrogliosis as detected by glial fibrillary acidic protein (GFAP) immunohistochemistry and confirmed with the analysis of its corresponding mean fluorescence intensity (MFI, Fig. 4). These effects were less intense in the rostral region, displaying 4.2 ± 0.8 healthy cells (78% loss) in the ipsilateral horn and 13.6 ± 1.0 (32% loss) in the contralateral side (Fig. 3b).

Rats co-infused with TTC and AMPA in the spinal cord tissue did not show differences in the number of MNs in the rostral region as compared to AMPA alone, but in the infused area we found a significant protection, counting 5.3 ± 0.9 MNs (74% loss) in the ipsilateral horn and 14.5 ± 0.7 (30% loss) in the contralateral side. However, both the pre and post i.m. injections of TTC protected substantially better against AMPA-induced neurodegeneration in both regions analyzed, with no significant differences between the pre and post treatments: the TTC pre-treatment preserved 6.8 ± 0.9/16.0 ± 0.8 MNs (ipsilateral/contralateral, respectively) in the infused area (66% and 25% loss, as compared to 88% and 47% with AMPA alone) and 10.4 ± 0.7/17.6 ± 0.6 MNs in the rostral region (47% and 14% loss, as compared to 78% and 32% with AMPA alone); after the TTC post-treatment the values were similar, with 6.5 ± 0.9/15.0 ± 0.5 MNs (ipsilateral/contralateral) in the infused segment and 9.4 ± 1.1/16.7 ± 0.6 in the rostral zone (Fig. 3). Although there was some increase in the GFAP labeling in these groups (AMPA + i.m. TTC), it was significantly less than the astrogliosis stimulated by AMPA alone (Fig. 4), indicating that TTC treatment decreases the astroglial response generated by AMPA. This protection correlates with the notable preservation of the motor behavior described above, which was also considerably greater after the i.m. treatments than after the spinal infusion of TTC.

### The blockade of Trk receptors in the muscle decreases the protective effect of TTC

**Figure 5:**
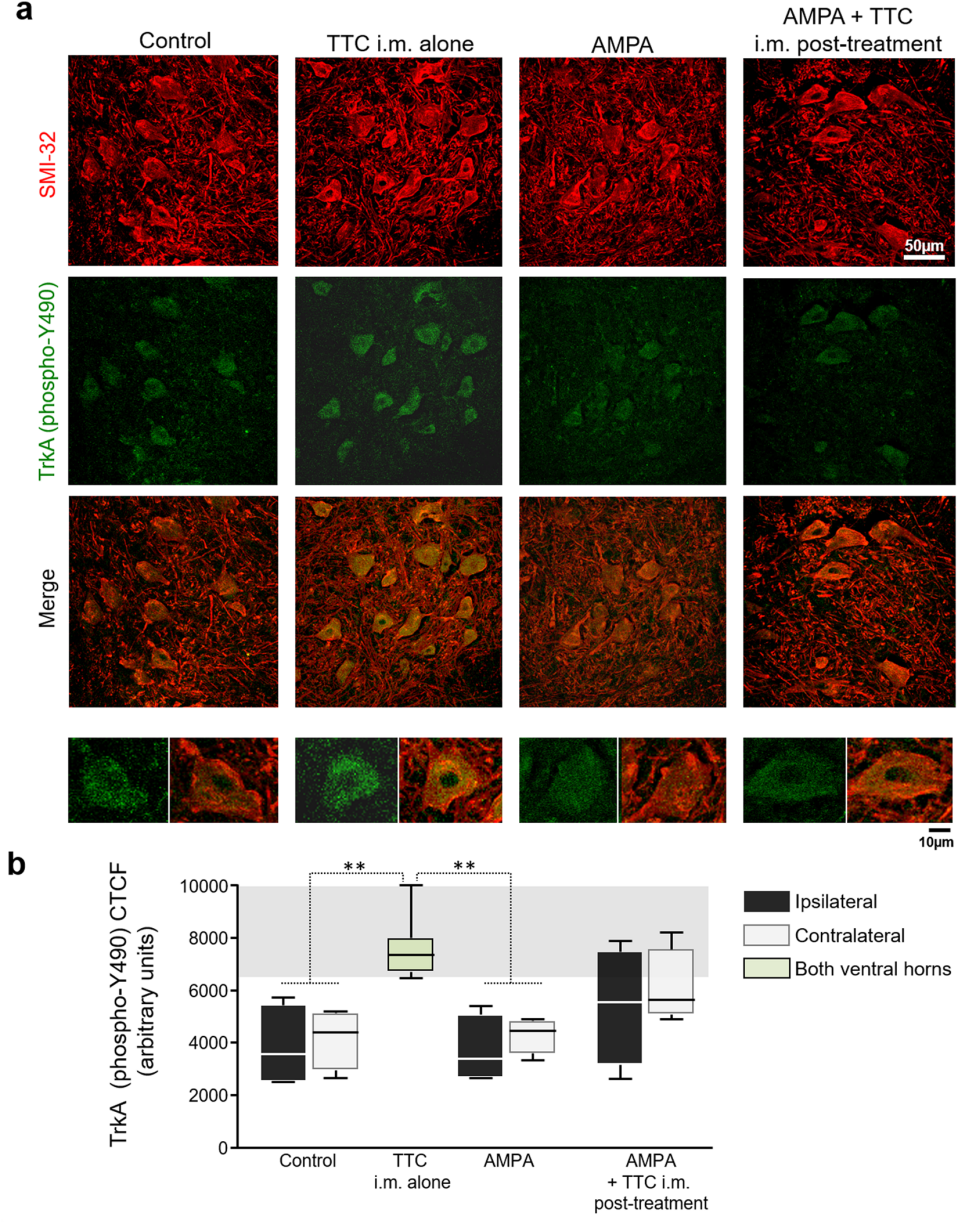
The i.m. administration of TTC increases TrkA receptor (phospho-Y490) in spinal MNs. (**a**) Representative confocal micrographs of the ipsilateral ventral horns immunostained for SMI-32 (red) and TrkA (phospho-Y490) (green) in the lumbar section of rats treated as indicated, four days after minipump implantation. The images on the bottom are magnifications of a representative MN with the TrkA (phospho-Y490) labeling (left) and the corresponding merged (right). (**b**) Quantitative analysis for TrkA (phospho-Y490) in spinal MNs. The TTC i.m. alone group received a bilateral i.m. administration of the fragment, and the green bar represents the average of both ventral horns. The analysis of the corrected total cell fluorescence (CTCF) was made using the maximum intensity projection of the Z-stack images for TrkA (phospho-Y490) alone. CTCF shows that TTC i.m. alone increased the phosphorylation of the TrkA receptor at Y490 in spinal MNs. Five histological slices/rat were analyzed (n = 4 per group). The boxes represent the 1st and 3rd quartile around the median. The whiskers above and below the box show the locations of the minimum and maximum values. The grey horizontal area highlights the maximum and minimum value for the AMPA group. **p < 0.01 vs the AMPA group.

Previous experiments *in vitro* have shown that the activation of Trk receptors is the onset of the signaling cascades triggered by TTC^33^. Until now, the contribution of each Trk member (A, B, C) in the TTC neuroprotective effect has not been detailed, but it is known that TTC is able to activate the TrkA receptor in synaptosomal preparation^34^. Therefore, to inquire the involvement of TrkA receptor in the protection exerted by TTC, we analyzed the presence of the activated phosphorylated (phospho-Y490) form of the receptor^43^ in the spinal cord, by means of immunofluorescence. A double immunohistochemistry for SMI-32 as a neuronal marker and for TrkA receptor (phospho-Y490) in control rats revealed co-localization of both indicators, thus confirming the presence, although not abundant, of this neurotrophic receptor in MNs (Fig. 5a), as has previously been reported using the same antibody^44^. AMPA treatment did not alter this puncta labeling for TrkA. However, as the micrographs and the quantitative analysis show, TTC i.m. alone increased the fluorescent signal for TrkA (phospho-Y490). The immunochemical analysis also revealed a tendency, although non-significant, of an increased labeling in the group AMPA + TTC i.m. post-treatment (Fig. 5b).

**Figure 6:**
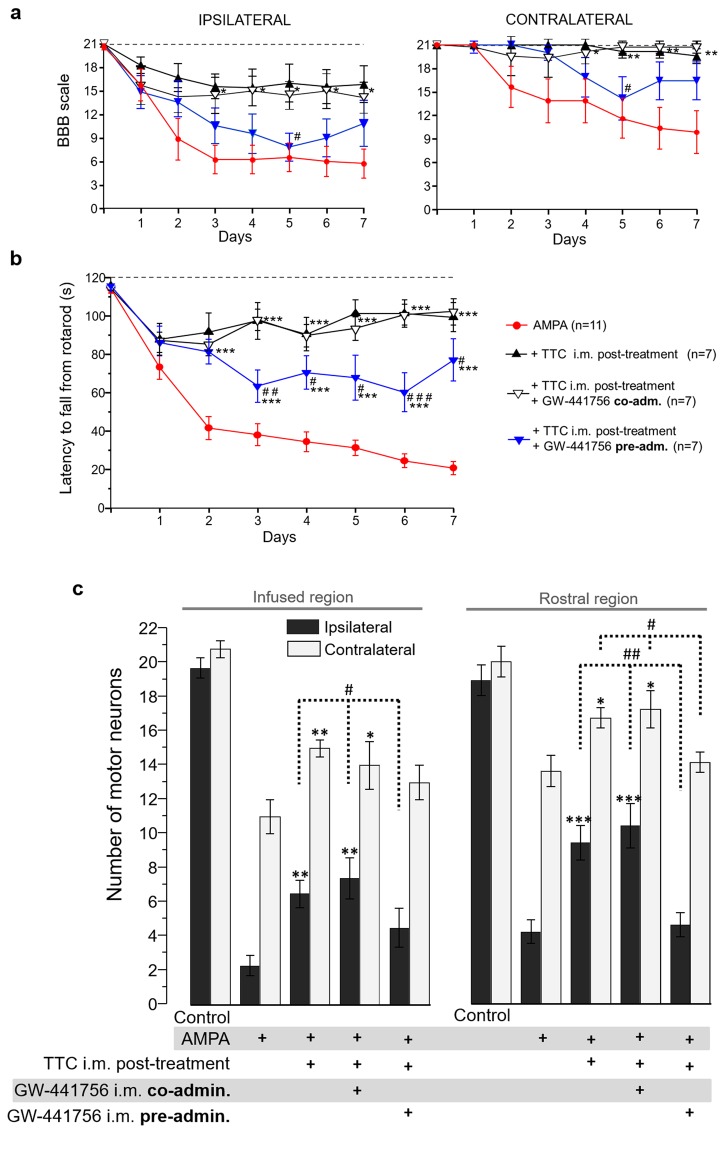
The i.m. administration of the TrkA receptor inhibitor GW-441756 prevents the protection exerted by TTC. The BBB locomotor rating scale for the ipsilateral and contralateral rear limb (**a**) as well as the motor performance assessed by the rotarod test (**b**) show that the i.m. administration of the inhibitor before the TTC, but not the co-administration, significantly decreased the protection induced by TTC. The top discontinuous line indicates the time registered by control animals. For the rotarod test three trials/day/rat were assessed. Data are mean values ± SEM for the number of rats shown in parentheses. p < 0.05, **p < 0.01, ***p < 0.001 vs AMPA and ^#^p < 0.05, ^##^p < 0.01, ^###^p < 0.001 vs the other TTC treatments. (**c**) Number of healthy MNs in the ipsilateral and contralateral ventral horns of the infused and rostral region of the lumbar spinal cord, seven days after minipump implantation. Fifteen histological slices/rat/region were analyzed. Data are mean values ± SEM for the number of rats indicated in (**a**-**b**). *p < 0.05, **p < 0.01, ***p < 0.001 vs corresponding side of the AMPA group in the corresponding region (infused or rostral). ^#^p < 0.05, ^##^p < 0.01 vs corresponding side of the GW-441756 i.m. pre-administration group.

Taking into account the effect of TTC on the TrkA receptor, and the fact that this receptor family has been described in the muscle^45,46^, we decided to study further the involvement of this receptor using GW-441756, an inhibitor of TrkA^47^. This drug was administered i.m. at different times, to test whether this treatment affected the protection by TTC (Fig. 1). In these experiments we studied only the effects of the i.m. TTC post-treatment, which was very effective even when the excitotoxic process had already started. The vehicle dimethyl sulfoxide (DMSO), alone or injected i.m. with GW-441756 was innocuous, as revealed by the lack of alterations in the morphology of MNs and in the motor tests used (data not shown). As shown in Fig. 6, the i.m. co-administration of TTC and GW-441756 did not alter the protection induced by the fragment neither in the BBB scale, the rotarod test nor in MN survival. However, when GW-441756 was injected 6 h and 24 h before TTC, significantly reduced, although not completely abolished, the protection exerted by the fragment. Figure 6a shows that the animals pre-administered with GW-441756 scored similarly to the AMPA group, with a slightly, although not significant, improvement in the last two days of evaluation. Rats of this group remained in the rotarod significantly less time (63.4 ± 8.5 s on day three and 77.0 ± 11.0 s on day seven) as compared to AMPA + TTC group (93.7 ± 9.5 s and 99.3 ± 7.6 s, respectively). This effect was evident from day 3 and lasted during the remaining days of evaluation (Fig. 6b). Correspondingly, this GW-441756 pre-administration reduced the protection by TTC against MN damage, since the MN counting in both the infused (4.5 ± 0.9/13.0 ± 1.0, ipsilateral/contralateral) and the rostral region (4.6 ± 0.8/14.1 ± 0.6) was comparable to that of the AMPA alone group (Fig. 6c).

## Discussion

In this work we demonstrate for the first time that the TTC i.m. treatment, with a 6–24 h effective time frame, remarkably protected against rear limbs paralysis and spinal MN death induced by the excitotoxic effect of the chronic AMPA infusion in the spinal cord, whereas the intraspinal infusion was less effective. The protection was partially prevented by the blockade of Trk receptors in the muscle.

The initial approach of infusing TTC in the spinal cord had the purpose to test a direct effect on the MNs cell bodies, but this strategy proved to be only slightly effective for protecting against AMPA-induced toxic effects. This result differs from data indicating that the direct application was protective against the neurodegeneration induced by 1-methyl-4-phenylpyridinium in the striatum^37^ and by β-amyloid peptide 25–35 in the medial septum^48^. This weak effect could be due to the constant transynaptic movement of the TTC and its concomitant clearance from the site of infusion^49,50^, but it could also be due to the scarce existence of its putative Trk receptors (A, B, C) in the ventral horn of the spinal cord^51,52^, which we partially corroborated in the present work by immunohistochemistry of the TrkA phosphorylated form (Fig. 5).

On the basis of our previous work^5^ we decided to inject the TTC into the gastrocnemius and the biceps femoris muscles, which correspond to the distal and proximal muscles innervated by the MNs of the spinal segments we infused^53^. The i.m. injection of the fragment, before or after the beginning of the AMPA-induced excitotoxic process, significantly decreased MN degeneration in both ventral horns and also diminished the GFAP labeling, findings that correlate with each other, as we have previously shown after AMPA-induced neurodegeneration^54^. In terms of functional outcomes, this protection resulted in a clear preservation of the movement of rear limbs, as demonstrated by the diminished alterations of the phalanges (better scores in the BBB scale) and improved performance of the rotarod test. We have previously shown that a small difference in the number of MNs in the segments of the lumbar spinal cord studied results in notable alterations in the motor skills, as assessed by the rotarod test^10^. Thus, we consider that this small difference in the number of healthy MNs is responsible of the better motor outcomes in the animals with i.m. TTC in comparison with spinal infusion group.

It is also important to remark that on day one the locomotor profile of the groups with TTC i.m. post-treatment is similar to the AMPA group, whereas the pre-treatment group show a decline until days 3–4. This difference could be related to the time required by TTC to reach its target receptor to finally generate a potent protective signaling, as mentioned below regarding the signaling endosome effect. A similar but much less significant protective effect has been described in the mutant SOD1-G93A murine model of familial ALS after the i.m. injection of naked DNA encoding for TTC^55^.

The TrkA receptor phosphorylation in Y490 is associated to the activation of survival pathways MAPK/ERK and PI3K^43^. Previous data from our group has shown that the activation of these routes by the direct administration of the vascular endothelial growth factor in the spinal cord was effective to protect against the toxic effect of AMPA in our chronic model of spinal MN degeneration^54^. Interestingly, the injection of TTC alone on the rear limb muscles was able to slightly increase this phosphorylation in spinal MNs, suggesting the involvement of this receptor in the mechanism of the protection. On this point, we tried to study if the presence of TTC was necessary in the spinal MNs to increase this TrkA phosphorylation; however, due to the lack of specificity of the antibody against the fragment we were not able to analyze it further (data not shown).

The effects of the inhibitor GW-441756 clearly indicate an involvement of Trk receptors in the protective action of TTC. The fact that its i.m. co-administration with TTC did not affect the protection induced by the fragment, whereas its i.m. pre-administration diminished the protection, as assessed by both the cellular and behavioral effects, suggests that that the action of TTC involves the TrkA receptor in the NMJ and that the inhibitor must bind to the receptor before TTC, although it could be related to the different receptor-ligand affinities or other pharmacodynamic factors. However, caution is necessary on this point because, although GW-441756 has been widely used as a specific inhibitor of TrkA receptor^47,56,57^, it has also been reported that the IC50 (*in vitro*) of GW-441756 for the three Trk receptors A, B, C are similar (0.23, 0.14, 0.46 µM respectively); this problem is shared by other Trk inhibitors such as AG879 (IC50: 1.3, 1.0, 1.3 µM respectively.) and K252A (0.13, 0.11, 0.30 µM)^58^. Thus, we cannot exclude the participation of the other isoforms (B, C) of the Trk receptor, as it has been proposed in studies *in vitro*^33^. The fact that some protection was still evident after the use of GW-441756 might be due to the possible participation of other binding sites for TTC at the NMJ, such as gangliosides^24,25^ and entactins^59^.

## Conclusion

On the basis of and the remarkable neuroprotective effect of the i.m. TTC treatment as compared with its intraspinal infusion, on the partial blockade of this protective action by the i.m. administration of the Trk receptors inhibitor, and on the extensively described retrograde transport of TTC *in vivo*^27,29,30,60,61^, we conclude that the effects of TTC are mediated by the retrograde transport of the fragment from muscle nerve terminals to spinal MNs. Interestingly, this sort of retrograde signaling-transport initiated at the neuromuscular terminals seems to be required also for the protection promoted by the TrkA-endosomes activated by neurotrophic factors^62–64^, which has been shown *in vivo*^65,66^.

Taking in consideration that TTC at low dosage is non-toxic and very efficient to exert neuroprotection when administered through a simple i.m. injection, even when it is given after the beginning of the excitotoxic degeneration process, we consider that this peptide has a potential therapeutic profile, but further studies are required to elucidate the details of the signaling mechanisms involved in the TTC-induced neuroprotection *in vivo*.

## Electronic supplementary material

Supplementary Table 1

## Data Availability

The datasets generated during and/or analyzed during the current study are available from the corresponding author on reasonable request.
