# Supplementary material for: Tetanus toxin C-fragment protects against excitotoxic spinal motoneuron degeneration in vivo
Source: Sci Rep. 2018 Nov 8;8:16584. doi: 10.1038/s41598-018-35027-w (PMC6224557; doi:10.1038/s41598-018-35027-w)
Supplement: Supplementary file 1 — Supplementary Table 1 [file 41598_2018_35027_MOESM1_ESM.pdf]

Supplementary Table 1.

Tetanus toxin C-fragment protects against excitotoxic spinal motoneuron degeneration in vivo. C. Netzahualcoyotzi and R. Tapia

The 21-Point Basso, Beattie, Bresnahan locomotor rating scale

Score		Locomotor characteristics
Normal	21	Consistent plantar stepping and coordinated gait, consistent toe clearance, predominant paw position is parallel throughout stance, consistent trunk stability, tail consistently up.
	20	Consistent plantar stepping and consistent coordinated gait; consistent toe clearance; predominant paw position is parallel at initial contact and lift off; tail consistently up; and trunk instability.
	19	Consistent plantar stepping and consistent no forelimb-hindlimb (FL-HL) coordination during gait; and toe clearance occurs consistently during forward limb advancement; predominant paw position is parallel at initial contact and lift off; and tail is down part or all of the time.
	18	Consistent plantar stepping and consistent FL-HL coordination during gait; and toe clearance occurs consistently during forward limb advancement; predominant paw position is parallel at initial contact and rotated at lift off.
	17	Consistent plantar stepping and consistent FL-HL coordination during gait; and toe clearance occurs frequently during forward limb advancement; predominant paw position is parallel at initial contact and lift off.
	16	Consistent plantar stepping and consistent FL-HL coordination during gait; and toe clearance occurs frequently during forward limb advancement; predominant paw position is parallel at initial contact and rotated at lift off.
	15	Consistent plantar stepping and consistent FL-HL coordination; and no toe clearance or occasional toe clearance during forward limb advancement; predominant paw position is parallel to the body at initial contact.
	14	Consistent weight supported plantar steps, consistent FL-HL coordination; and predominant paw position during locomotion is rotated (internally or externally) when it makes initial contact with the surface as well as just before it is lifted off at the end of stance or frequent plantar stepping, consistent FL-HL coordination, and occasional dorsal stepping.
	13	Frequent to consistent weight supported plantar steps and frequent FL-HL coordination.
	12	Frequent to consistent weight supported plantar steps and occasional FL-HL coordination.
	11	Frequent to consistent weight supported plantar steps and no FL-HL coordination.
	10	Occasional weight supported plantar steps, FL-HL coordination.
	9	Plantar placement of the paw with weight support in stance only (i.e., when stationary) or occasional, frequent, or consistent weight supported dorsal stepping and no plantar stepping.
	8	Sweeping with no weight support or plantar placement of the paw with no weight support.
	7	Extensive movement of all three joints of the HL.
	6	Extensive movement of two joints and slight movement of the third.
	5	Slight movement of two joints and extensive movement of the third.
	4	Slight movement of all three joints of the HL.
	3	Extensive movement of two joints.
	2	Extensive movement of one joint or extensive movement of one joint and slight movement of one other joint.
	1	Slight movement of one or two joints (hip and/or knee).
Paralysis	0	No observable HL movement.
